# QSAR-Driven Design and Discovery of Novel Compounds With Antiplasmodial and Transmission Blocking Activities

**DOI:** 10.3389/fphar.2018.00146

**Published:** 2018-03-06

**Authors:** Marilia N. N. Lima, Cleber C. Melo-Filho, Gustavo C. Cassiano, Bruno J. Neves, Vinicius M. Alves, Rodolpho C. Braga, Pedro V. L. Cravo, Eugene N. Muratov, Juliana Calit, Daniel Y. Bargieri, Fabio T. M. Costa, Carolina H. Andrade

**Affiliations:** ^1^LabMol – Laboratory for Molecular Modeling and Drug Design, Faculty of Pharmacy, Federal University of Goiás, Goiânia, Brazil; ^2^Laboratory of Tropical Diseases – Prof. Dr. Luiz Jacintho da Silva, Department of Genetics, Evolution, Microbiology and Immunology, Institute of Biology, UNICAMP, Campinas, Brazil; ^3^Laboratory of Cheminformatics, PPG-SOMA, University Center of Anápolis/UniEVANGELICA, Anápolis, Brazil; ^4^Global Health and Tropical Medicine Centre, Unidade de Parasitologia Médica, Instituto de Higiene e Medicina Tropical, Universidade Nova de Lisboa, Lisbon, Portugal; ^5^Laboratory for Molecular Modeling, Division of Chemical Biology and Medicinal Chemistry, Eshelman School of Pharmacy, University of North Carolina at Chapel Hill, Chapel Hill, NC, United States; ^6^Department of Chemical Technology, Odessa National Polytechnic University, Odessa, Ukraine; ^7^Department of Parasitology, Institute of Biomedical Sciences, University of São Paulo, São Paulo, Brazil

**Keywords:** malaria, virtual screening, QSAR, *Plasmodium falciparum*, dUTPase, transmission blocker

## Abstract

Malaria is a life-threatening infectious disease caused by parasites of the genus *Plasmodium*, affecting more than 200 million people worldwide every year and leading to about a half million deaths. Malaria parasites of humans have evolved resistance to all current antimalarial drugs, urging for the discovery of new effective compounds. Given that the inhibition of deoxyuridine triphosphatase of *Plasmodium falciparum* (*Pf*dUTPase) induces wrong insertions in plasmodial DNA and consequently leading the parasite to death, this enzyme is considered an attractive antimalarial drug target. Using a combi-QSAR (quantitative structure-activity relationship) approach followed by virtual screening and *in vitro* experimental evaluation, we report herein the discovery of novel chemical scaffolds with *in vitro* potency against asexual blood stages of both *P. falciparum* multidrug-resistant and sensitive strains and against sporogonic development of *P. berghei*. We developed 2D- and 3D-QSAR models using a series of nucleosides reported in the literature as *Pf*dUTPase inhibitors. The best models were combined in a consensus approach and used for virtual screening of the ChemBridge database, leading to the identification of five new virtual *Pf*dUTPase inhibitors. Further *in vitro* testing on *P. falciparum* multidrug-resistant (W2) and sensitive (3D7) parasites showed that compounds LabMol-144 and LabMol-146 demonstrated fair activity against both strains and presented good selectivity versus mammalian cells. In addition, LabMol-144 showed good *in vitro* inhibition of *P. berghei* ookinete formation, demonstrating that hit-to-lead optimization based on this compound may also lead to new antimalarials with transmission blocking activity.

## Introduction

Malaria is an infectious disease caused by protozoans of the genus *Plasmodium* and transmitted through the bite of insect vectors of the genus *Anopheles. Plasmodium falciparum* is the most prevalent and lethal species infecting humans in the African continent, being responsible for 99% of all malaria-attributed deaths (World Health Organization [WHO], 2016). Despite the fact that integrated control interventions have achieved significant progress in the reducing malaria cases and related mortality in recent years, malaria still causes 429,000 deaths every year, being endemic in 91 countries and territories of sub-Saharan Africa, South-East Asia, Latin America, and the Middle East (World Health Organization [WHO], 2016).

When compared to viruses and bacteria, these eukaryotic protozoans present a larger genome, have multiple stages in their life cycle, and a complex biology, which hinder the development of vaccines (Hoffman et al., 2015). Consequently, malaria control strategies largely rely on drug-dependent case management. Currently, artemisinin-based combination therapy (ACT) is the recommended official treatment for malaria. However, resistance to artemisinins has been detected in five countries in the Greater Mekong sub region of South-east Asia, endangering the future of *P. falciparum* elimination (Vogel, 2014; World Health Organization [WHO], 2016; Thu et al., 2017). Therefore, there is an urgent need for the discovery and development of new antimalarial therapies.

The enzyme 2′-deoxyuridine 5′-triphosphate nucleotide hydrolase (dUTPase) has emerged as a promising biological target in *P. falciparum*, and it is responsible for the hydrolytic cleavage of dUTP (deoxyuridine triphosphate) in dUMP (deoxyuridine monophosphate) and pyrophosphate (Nyman, 2001). The inhibition of dUTPase may cause dUTP accumulation and erroneous incorporation of uracil into DNA, leading to parasite death. Although another enzyme, DNA glycosylase, could repair the erroneous insertions, the excessive number of repairs would result in a fatal break of DNA strand (Whittingham et al., 2005). Given that DNA replication in *Plasmodium* takes place in all distinct stages of the parasite life cycle and given the importance of the enzyme dUTPase in this process, this enzyme is expressed in both asexual and sexual stages of the parasite (ring, trophozoite, schizont, gametocyte, and ookinete), as demonstrated in previous studies on *P. falciparum* 3D7 and *P. berghei* (López-Barragán et al., 2011; Otto et al., 2014). Thus, dUTPase inhibitors might not only act against blood-stage parasites, but also could block parasite transmission/development in mosquitoes. Experimental findings categorize dUTPase as essential for various organisms, such as *Escherichia coli, Saccharomyces cerevisiae, and Mycobacterium smegmatis* (El-hajj et al., 1988; Gadsden et al., 1993; Pecsi et al., 2012). The dUTPase of *P. falciparum* (*Pf*dUTPase) is an attractive target for the development of selective inhibitors since it presents relatively low sequence similarity with its human ortholog *Hs*dUTPase (28.4% identity) (Whittingham et al., 2005).

Due to the importance of dUTPase in the parasite’s DNA repair, we decided to use computer-aided drug design (CADD) approaches for discovering new dUTPase inhibitors. In the last several decades, CADD approaches have been widely applied in early stages of drug discovery, making the process faster and more financially viable (Leelananda and Lindert, 2016). Among these approaches, quantitative structure-activity relationships (QSARs) have been extensively used for lead optimization and virtual screening (Verma et al., 2010). Different QSAR approaches have been used by our group for identification of new promising hits for infectious diseases (Melo-Filho et al., 2016; Neves et al., 2016; Gomes et al., 2017).

In this work, we applied a combi-QSAR approach, combining 2D- and 3D-QSAR models, in a virtual screening campaign of the ChemBridge database for selection of new antimalarial virtual hits. Finally, we performed *in vitro* experimental evaluation of the potential *Pf*dUTPase inhibitors against chloroquine-sensitive and multidrug-resistant strains of *P. falciparum*, and in gametocyte to ookinete conversion of *P. berghei*, aiming to identify new potential and selective antimalarial hits.

## Materials and Methods

**FIGURE 1:**
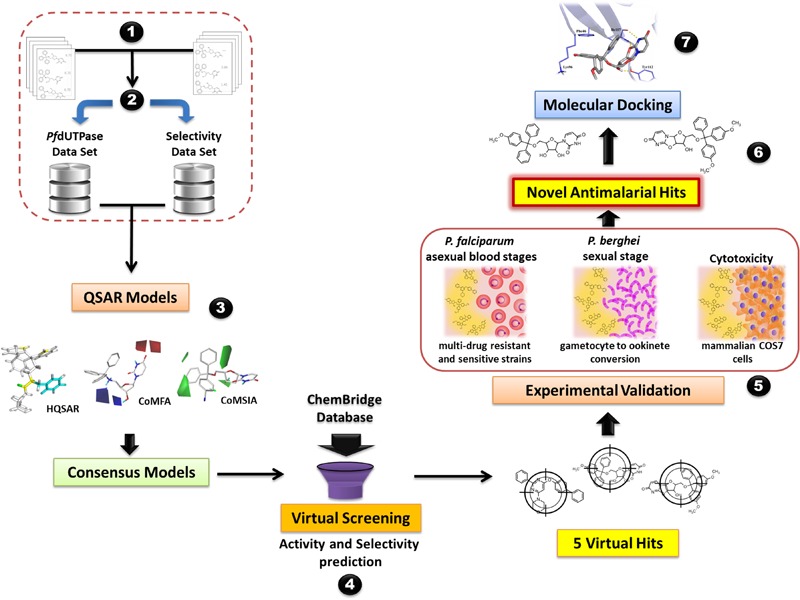
General workflow of the computer-aided design and discovery of new antimalarial hits using Combi-QSAR models for virtual screening followed by experimental validation. Briefly, the following steps were performed: (1) data integration and compilation; (2) data curation; (3) QSAR models generation and validation; (4) virtual screening of the ChemBridge database and selection of the compounds with higher predicted potency and selectivity; (5) experimental validation against *P. falciparum* asexual blood stages, *P. berghei* sexual stage, and mammalian COS7 cells; (6) identification of novel antimalarial hits; (7) molecular docking of the most promising antimalarial hit in plasmodial and human dUTPase.

The steps of the modeling study are briefly presented in **Figure 1**. The workflow encompasses the following steps: (i) data compilation and integration; (ii) data curation; (iii) model generation; (iv) virtual screening and (v) experimental validation. Our workflow was built following the best practices of QSAR modeling and CADD (Tropsha, 2010; Cherkasov et al., 2014).

### Dataset Preparation

2D and 3D QSAR models were built using a series of *Pf*dUTPase inhibitors reported in the literature (Supplementary Table S1) (Nguyen et al., 2005, 2006; Whittingham et al., 2005; McCarthy et al., 2009; Baragaña et al., 2011; Hampton et al., 2011; Ruda et al., 2011). The data set was prepared and curated according to the protocol described by Fourches et al. (2010, 2015, 2016). Counterions were removed as chemotypes, and specific and nitroaromatic groups were standardized using Standardizer (v. 6.1, ChemAxon, Budapest, Hungary^1^). Duplicates were identified using ISIDA Duplicates program (Varnek et al., 2008) and HiTQSAR (Kuz’min et al., 2008). If values of properties of identical compounds were equal, one of these compounds was kept in the data set. However, if properties were significantly different, all records were removed. After curation, 127 compounds (Supplementary Table S1) with activity against *Pf*dUTPase were kept for molecular modeling. The activity against both *Plasmodium* and human enzymes was available only for 45 compounds and used for calculation of selectivity (*S*) (Eq. 1). The activity was represented as *K*_i_ (inhibition constant) and converted to the corresponding p*K*_i_ (-log*K*_i_). In a similar approach, selectivity was converted to the logarithmic scale:

(1) $$S=\log\frac{HsdUTPaseKi}{PfdUTPaseKi}$$

Values of *S* greater than zero indicate selective compounds while values below zero indicate compounds with poor selectivity.

The data sets were divided into training and test sets using the Hierarchical Cluster Analysis method (HCA) available in the SYBYL v.1.2 (SYBYL-X 1.2, Tripos International, St. Louis, MO, United States). Molecules representing each cluster were manually selected for test set to maximize the coverage across the entire range of inhibition activity and selectivity. The final proportion between training and test set compounds was 3:1.

### HQSAR

Hologram QSAR (HQSAR), available on SYBYL-X v.1.2 (SYBYL-X 1.2, Tripos International, St. Louis, MO, United States; TRIPOS, 2010a), was used to build 2D QSAR models. Holograms were generated using six distinct fragment sizes (2–5, 3–6, 4–7, 5–8, 6–9, 7–10 atoms) over a series of hologram lengths (53–997). Different combinations of fragment distinction were also considered, such as atoms (A), bonds (B), connectivity (C), hydrogen atoms (H), chirality (Ch), and hydrogen bond donor/acceptor (DA).

### Conformer Generation and Atomic Charges Assignment

The structures were converted into 3D format, and initial conformations were generated using the OMEGA v.2.5.1.4 (Hawkins et al., 2010; OMEGA 2.5.1.4: OpenEye Scientific Software, Santa Fe, NM, United States^2^). Two different methods were used for the calculation of the partial atomic charges: the empirical method of Gasteiger-Hückel available on SYBYL-X v.1.2 (SYBYL-X 1.2, Tripos International, St. Louis, MO, United States) and the semi-empirical AM1-BCC (Jakalian et al., 1999, 2002) implemented in QUACPAC v.1.6.3.1 (QUACPAC 1.6.3.1: OpenEye Scientific Software, Santa Fe, NM, United States^2^). The protonation state of the molecules were performed at pH 7.4, using QUACPAC 1.6.3 (QUACPAC 1.6.3.1: OpenEye Scientific Software, Santa Fe, NM, United States^2^).

### Molecular Alignment

Compounds were submitted to three different molecular alignments: (i) alignment based on the morphological similarity function implemented in Surflex-Sim, accessible in SYBYL-X 1.2 (SYBYL-X 1.2, Tripos International, St. Louis, MO, United States); (ii) shape-based alignment from ROCS 3.2.1.4 software (Hawkins et al., 2007; ROCS 3.2.1.4: OpenEye Scientific Software, Santa Fe, NM, United States^2^); and (iii) alignment by molecular docking of molecules on *Pf*dUTPase, using OEDocking 3.0.1 software (OEDocking 3.2.0.2: OpenEye Scientific Software, Santa Fe, NM, United States^2^). For the last alignment, X-ray crystal structure of *Pf*dUTPase complexed with the inhibitor 2′,5′-dideoxy-5′-[(diphenylmethyl)amino]uridine (PDB ID: 3T64) (Hampton et al., 2011) was imported to Maestro v. 9.3 (Epik version 3.0, Schrödinger, LLC, New York, NY, United States, 2014.) and prepared using Protein Preparation Wizard, where hydrogen atoms were added according to Epik v. 2.7 (Epik version 3.0, Schrödinger, LLC, New York, NY, United States, 2014.; Shelley et al., 2007) (pH 7.4 ± 0.5), and minimized using the OPLS-2005 force field (Banks et al., 2005). On Make Receptor tool, available on OEDocking 3.0.1 (OEDocking 3.2.0.2: OpenEye Scientific Software, Santa Fe, NM, United States^2^), the receptor grid was generated with dimensions 22.34 Å × 19.65 Å × 25.24 Å and volume of 11,078 Å^3^. All compounds of the data set were docked and the best pose for each molecule was selected for alignment.

### 3D-QSAR

Comparative Molecular Field Analysis (CoMFA) and Comparative Molecular Similarity Indices Analysis (CoMSIA), available in SYBYL-X v.1.2 (SYBYL-X 1.2, Tripos International, St. Louis, MO, United States; TRIPOS, 2010b), were used to build 3D QSAR models for *Pf*dUTPase inhibitors.

#### CoMFA

The aligned training set molecules were placed in a 3D lattice box with grid spacing of 2 Å. Then, CoMFA steric and electrostatic fields were calculated at each grid point with the Tripos force field using a carbon atom probe with sp^3^ hybridization (Csp^3^) and charge +1.0. The energy cutoff was set to 30 kcal/mol. The standard deviation coefficient method (SDC) was used for region focusing with values varying from 0.3 to 1.5.

#### CoMSIA

The models were generated using the same molecular alignments used for CoMFA. The aligned compounds were placed in the 3D lattice box with grid spacing of 2 Å. The steric, electrostatic, hydrophobic, hydrogen bond donor and acceptor descriptors were calculated at each grid point. A probe carbon atom with radius of 1.0 Å and charge +1.0, was used to obtain the similarity indices. A Gaussian function was used to describe the energy terms according to the distance between the probe atom and aligned molecules. The attenuation factor (α) was used on default value of 0.3.

### Generation and Validation of QSAR Models

Partial least squares regression (PLS) was used for development of statistical models (Lindberg et al., 1983). The internal validation of QSAR models was performed using the full cross-validation *r*^2^ (*q*^2^) leave-one-out (LOO) method. The predictive ability of the models was assessed by *Q*^2^_ext_ (Tropsha et al., 2003) estimated on external set compounds that were not used for model building or selection. The consensus models were obtained by combination of three QSAR models (HQSAR + CoMFA + CoMSIA). The models were built and used separately for predictions. The predicted activity of each compound by the consensus model was the result of the arithmetic mean of individual models predictions. The external validation of these models was done using the same metrics as for individual models.

### Virtual Screening

The virtual screening of new potential *Pf*dUTPase inhibitors was performed on Hit2Lead library of the ChemBridge database (ChemBridge Online Chemical Store, 2017). All compounds were prepared using the same protocol and software used in the preparation of the modeling dataset. The methods of alignment and partial charges calculation were the same used in the best individual CoMFA and CoMSIA models. Then compounds had their activity and selectivity predicted by the consensus QSAR models. Two criteria were used for selection of virtual hits: (i) compounds should have the highest predicted potency against *Pf*dUTPase (predicted p*K*_i_); (ii) the predicted selectivity (*S*) should be greater than zero. Furthermore, some ADMET properties were predicted for the best virtual hits, such as physicochemical properties (log*P* and log*S*)^3^), acute oral toxicity by GUSAR^4^ (Filimonov et al., 2004; Lagunin et al., 2009, 2011), carcinogenicity using admetSAR^5^ (Cheng et al., 2012), and hERG K+ channel blockage using Pred-hERG^6^) (Alves et al., 2014; Braga et al., 2014, 2015).

### Molecular Docking

The selected virtual hits were submitted to molecular docking in Glide (Friesner et al., 2004), available on Maestro v. 9.3.5, to predict their binding mode in *Pf*dUTPase and human dUTPase (*Hs*dUTPase). Ligands were prepared on LigPrep module of Maestro software, the correct protonation states and energy minimization were performed on Epik v. 2.7 (pH 7.4 ± 2.0) using OPLS-2005 force field. The previously prepared structure of *Pf*dUTPase, used for docking-based alignment, was used here. The search space was defined as a box with 10 x 10 x 10 Å^3^. The box was centered on the geometrical center of co-crystallized ligand (-7.7431 Å × 27.0662 Å ×-3.9483 Å, *x, y* and *z* axes, respectively). The structure of *Hs*dUTPase (PDB ID: 3ARA, resolution of 1.7 Å) (Miyakoshi et al., 2012) was prepared using the same protocol described for plasmodial enzyme. The grid was defined with dimensions 10 × 10 × 10 Å^3^ and was centered on the co-crystallized ligand at 6.3901 Å × 11.1138 Å ×-17.3607 Å, *x, y* and *z* coordinates. After docking, the poses of each virtual hit were submitted to rescoring using the Molecular Mechanics/Generalized Born Surface Area (MM-GBSA) approach, available on Prime v.3.1 (Prime version 3.1, Schrödinger, LLC, New York, NY, United States, 2014), using default conditions.

### Experimental Evaluation

#### *Plasmodium* Culture

Chloroquine-sensitive (3D7) and multidrug-resistant (W2) strains were cultured in RPMI 1640 medium supplemented with 0.05 mg/mL gentamycin, 38.4 mM HEPES, 0.2% sodium bicarbonate, and 10% O^+^ human serum, as previously described in a standardized protocol (Trager and Jensen, 1976). Then, erythrocytes were added to the culture to obtain a 5% hematocrit, and incubated at 37°C under 5% CO_2_ atmosphere, with daily exchange of medium. The parasitemia was monitored daily in smears stained with Giemsa. Synchronic cultures in the ring stage were obtained by two consecutive treatments at 48 h intervals with a 5% solution of D-sorbitol (Lambros and Vanderberg, 1979).

#### Determination of Growth Inhibition by SYBR Green I

Parasites synchronized at the ring stage, with 0.5% parasitemia and 2% hematocrit were distributed in each well, separately. The compounds were tested in triplicates, using 12 point of concentration, prepared in two-fold dilution (40 μM – ∼0.019 μM) over 72 h. Chloroquine and pyrimethamine were used as control. Subsequently, the *in vitro* susceptibility of parasite to tested drugs was measured by SYBR Green according to Hartwig et al. (2013). Briefly, 100 μL of lysis buffer (20 mM Tris, 5 mM EDTA, 0.008% wt/vol saponin, 0.08% vol/vol Triton X-100, and 0.4 μL/mL of SYBR Green) were added in each well of a new black 96-well plate and 100 μL of parasite culture incubated with drugs were added. After homogenization, the plates were incubated for 1 h in the dark. Fluorescence was measured at 490 nm excitation and 540 nm emission (CLARIOstar, Labtech BMG). The IC_50_ was calculated by plotting the Log doses vs. Inhibition (expressed as a percentage relative to the control) in Prism 6 (GraphPad Software Inc.).

#### Cytotoxicity Assay

Cytotoxicity assays used COS7 cells (fibroblast-like cell lines derived from monkey kidney tissue), grown in DMEM medium supplemented with 10% fetal bovine serum and 0.05 mg/mL gentamicin in atmosphere containing 5% CO_2_ at 37°C. Drug cytotoxicity in COS7 cells was determined in duplicate, using 12 point of concentration, prepared in two-fold dilution (200 μM – ∼ 0.097 μM). After the incubation period (72 h), the cell viability analysis were done by the MMT reduction method (3-[4,5-dimethyl-thiazol-2-yl]-2,5-diphenyltetrazolium chloride (Mosmann, 1983). The optical density was determined at 570 nm (CLARIOstar, Labtech BMG) and the 50% cytotoxicity concentrations (CC_50_) was expressed as the percent viability relative to the control. The selectivity index of the compounds was determined by the following expression:

(2) $$S=\frac{COS7CC_{50}}{PfIC_{50}}$$

Where COS7 CC_50_ corresponds to the 50% cytotoxic concentration on COS7 cells and *Pf* IC_50_ is the 50 % inhibitory concentration on *P. falciparum* (3D7).

#### Ookinete Assay

All animal procedures were carried out in accordance to the Brazilian College of Animal Experimentation (COBEA). This research protocol was approved by the Ethics Committee of the Institute of Biomedical Sciences – University of Sao Paulo, protocol number 132/2014-CEUA. C57BL/6 mice received an intraperitoneal injection of *P. berghei* ANKA infected erythrocytes, and four days after infection, a mouse with parasitemia between 4 and 6% and gametocytemia > 0.4% was selected as blood donor for cardiac puncture. Four microliters of the infected blood was dispensed in 80 μl of ookinete medium (Blagborough et al., 2012) at 21°C with DMSO control or with 10 μM of the tested compounds. The assay was incubated at 21°C for 24 h and 2 μl of the blood at the bottom of the tubes was spread onto a glass slide, stained with Giemsa and analyzed under a direct light microscope. The total number of formed ookinetes were counted in each slide (triplicate for each condition), and inhibition was calculated in relation to the total ookinetes formed in the control condition.

## Results and Discussion

### QSAR Modeling

Various combinations of hologram length, fragment size, and fragment distinction were tested with an aim to build robust and predictive HQSAR models. The original data set was divided into training and test sets in a ratio of approximately 3:1 using the HCA method. The three best HQSAR models for *Pf*dUTPase inhibition are shown in Supplementary Table S2. The models displayed very similar statistical features, but the model with fragment distinction A/C (Supplementary Table S2) performed slightly better than others in terms of robustness (*q*^2^_LOO_ = 0.70) and external predictivity (*Q*^2^_ext_ = 0.71). In addition, the best model presented a Durbin-Watson metric (Savin and White, 1977) (*d*) closest to the ideal value (*d* = 1.99), indicating that this model is less biased. The Durbin-Watson test is useful to evaluate the presence or absence of autocorrelation of residuals from regression analysis. The values range from 0 to 4. Values of *d* near or equal to 2 indicate no autocorrelation of residuals. Values of *d* < 2 or *d* > 2 indicate that residuals are positively or negatively auto correlated and predictions are more biased (Savin and White, 1977). The best HQSAR models for selectivity (using human dUTPase data) are also presented in Supplementary Table S2. The best model, with fragment distinction B/C (Supplementary Table S2), showed good external predictivity (*Q*^2^_ext_ = 0.83), with *d*-value close to the reference value (*d* = 2.02). The plots comparing the experimental and predicted biological activity for the best HQSAR models are shown in Supplementary Figures S1A,D. These plots demonstrate a good agreement between experimental data and predictions from the models.

**FIGURE 2:**
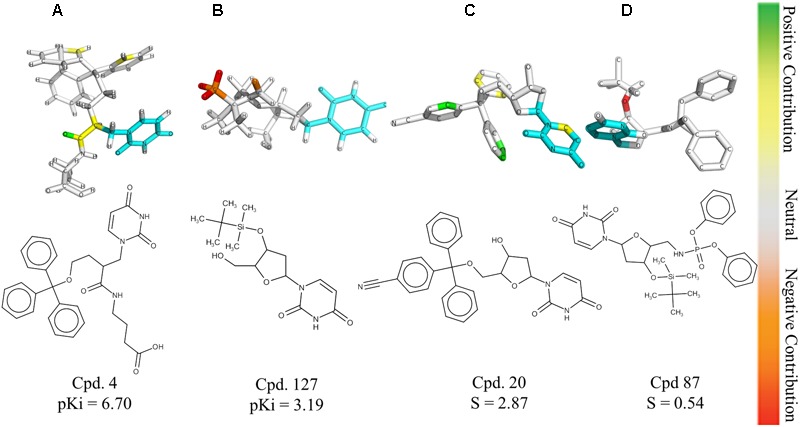
HQSAR contribution maps for the most potent *Pf*dUTPase inhibitor of the dataset (**A**, Cpd. 4) and the less potent compound (**B**, Cpd. 127). The most selective (**C**, Cpd. 20) and less selective (**D**, Cpd. 87) compounds are also displayed. The uracil ring, which is the common substructure, is highlighted in cyan.

The HQSAR contribution maps are useful to highlight the relationships between specific structural fragments and the biological property/activity. Colors close to the red end (red, red orange, and orange) indicate fragments with negative contribution, while colors in the green region (yellow, green blue, and green) indicate fragments with positive contribution to biological activity. The common substructure is represented in cyan (**Figure 2**).

The contribution maps of the most potent (4) and least potent inhibitors (127) and of the most selective (20) and least selective inhibitor (87) are presented in **Figure 2**. As one can see, the trityl ring has a positive contribution for both inhibition and selectivity (compounds 4 and 20, **Figure 2**). Additionally, the absence of the trityl group results in drastic decrease in activity against *Pf*dUTPase, as observed in compounds 4 and 127 (**Figures 2A,B**, respectively), and a clear decrease in selectivity, when we compare compounds 20 and 87 (**Figures 2C,D**, respectively). These observations corroborate previous studies (Whittingham et al., 2005; McCarthy et al., 2009; Baragaña et al., 2011; Hampton et al., 2011; Recio et al., 2011; Ruda et al., 2011; Ojha and Roy, 2013), indicating that two of the three phenyl rings from the trityl group have significant interactions with the hydrophobic pocket formed by residues Phe46 and Ile117 from *Pf*dUTPase (Hampton et al., 2011). In contrast, in the human enzyme, such residues are replaced by hydrophilic residues Val42 and Gly87. Therefore, there is no corresponding hydrophobic pocket in *Hs*dUTPase (Whittingham et al., 2005; Hampton et al., 2011). In a previous study by Ojha and Roy (2013), some nucleoside inhibitors were used for QSAR studies and pharmacophore mapping of *Pf*dUTPase inhibitors. The results revealed that two phenyl rings from the trityl group are responsible for stablishing important hydrophobic interactions and one phenyl ring may form a π–π stacking interaction with the amino acid residue Phe46 from *Pf*dUTPase (Ojha and Roy, 2013).

Two steps are critical for the development of CoMFA and CoMSIA models: the partial atomic charge assignment and structural alignment (Doweyko, 2004; Melo-Filho et al., 2014). In this study, two different charges (Gasteiger-Hückel and AM1-BCC) and three different molecular alignment approaches (morphological similarity function on Surflex-Sim, shape-based superposition on ROCS and alignment accessed by molecular docking) were evaluated. The Surflex-Sim alignment was performed using the most potent inhibitors of the data set (compounds 1 and 2) as templates, which were used for the flexible alignment of the remaining compounds of the data set. The shape-based alignment was executed with previously generated conformers. These conformers were superimposed to compound 3, which is the co-crystallized inhibitor of *Pf*dUTPase, available at Protein Data Bank (PDB code: 3T64) (Hampton et al., 2011). The superposition was evaluated by the *TanimotoCombo* score (Hawkins et al., 2010). Based on this score, the best conformation of each compound was selected. In the docking-based alignment, the previously generated conformers were docked and classified using the Chemgauss4 score function (McGann, 2011). The best conformer for each compound was selected based on the Chemgauss4 score. Additionally, conformers were visually inspected for selection of those with better superposition to the co-crystallized inhibitor.

The results of the best CoMFA and CoMSIA models are available at Supplementary Tables S3 and S4, respectively. The plots comparing the experimental and predicted biological activity for the best COMFA and CoMSIA models are shown in Supplementary Figures S1B,C,E,F. The best CoMFA models for inhibition and selectivity presented good robustness (*q*^2^_LOO_ = 0.63 and 0.86, respectively) and good external predictivity (*Q*^2^_ext_ = 0.75 and 0.61). Furthermore, presented good *d* values, indicating a low probability of biased predictions (*d* = 1.86 and 1.99, respectively). In general, for CoMFA models, the shape-based and Surflex-Sim alignments performed better than the docking-based alignment (Supplementary Table S3). The best CoMSIA models were obtained using shape-based alignment and AM1-BCC charges (Supplementary Table S4). The best CoMSIA model for *Pf*dUTPase inhibition presented good robustness and external predictivity (*q*^2^_LOO_ = 0.68; *Q*^2^_ext_ = 0.78, Supplementary Table S4). The best CoMSIA model for selectivity, despite its lower internal consistence (*q*^2^_LOO_ = 0.59), presented an acceptable external predictivity (*Q*^2^_ext_ = 0.63), as demonstrated on Supplementary Table S4.

**FIGURE 3:**
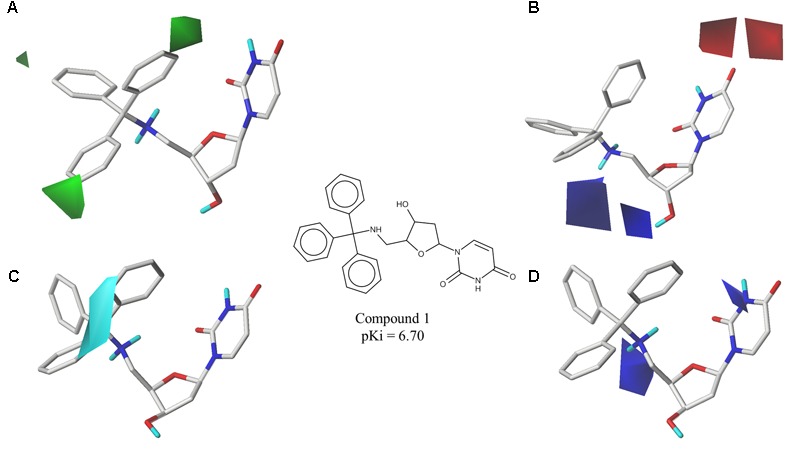
Contour maps of the best CoMFA and CoMSIA models for *Pf*dUTPase inhibition surrounding the most potent inhibitor (cpd. 1); **(A,B)** CoMFA steric and electrostatic contour maps; **(C,D)** CoMSIA electrostatic and hydrophobic contour maps. Steric fields: green contours indicate regions where bulky groups are favorable to biological activity; electrostatic fields: red contours indicate regions where electronegative groups are favorable for biological activity, while blue contours indicate regions where electronegative groups are unfavorable; hydrophobic fields: cyan contours indicate regions where hydrophobic groups are favorable to biological activity.

**FIGURE 4:**
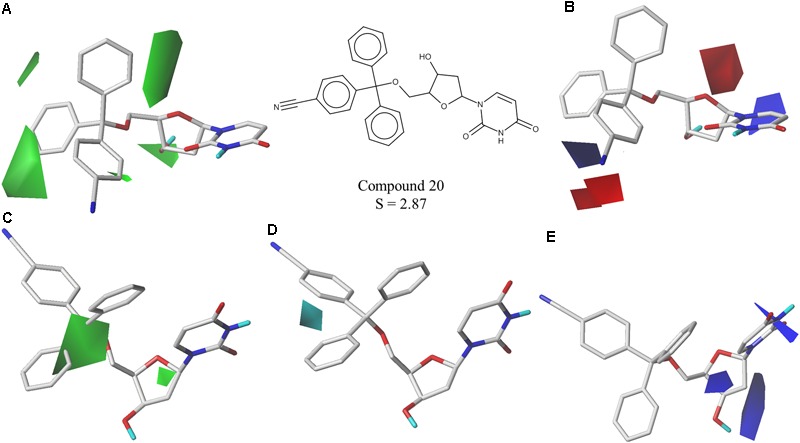
Contour maps of the best CoMFA and CoMSIA models for selectivity surrounding one of the most selective inhibitors (cpd. 20); **(A,B)** CoMFA steric and electrostatic contour maps; **(C–E)** CoMSIA steric, hydrophobic and electrostatic contour maps. Steric fields: green contours indicate regions where bulky groups are favorable to selectivity; electrostatic fields: red contours indicate regions where electronegative groups are favorable to selectivity, while blue contours indicate regions where electronegative groups are unfavorable; hydrophobic fields: cyan contours indicate regions where hydrophobic groups are favorable to selectivity.

The best CoMFA and CoMSIA models were used to generate contour maps by using STDEV^∗^COEFF field type and the function “contour by actual.” These maps could be useful for designing new potent and selective *Pf*dUTPase inhibitors as they indicate regions in the molecules where certain types of interactions are favorable and unfavorable for biological activity. The contour maps from the best CoMFA and CoMSIA models, for both inhibition and selectivity, are presented in **Figures 3, 4**, respectively.

The obtained contour maps show that bulky and hydrophobic groups in the trityl group region are favorable for both *Pf*dUTPase inhibition and selectivity (**Figures 3A,C, 4A,C,D**). These results corroborate with the HQSAR contribution maps and other studies highlighting the importance of the trytil hydrophobic group for inhibition and selectivity. The trytil group interacts with the hydrophobic pocket formed by residues Phe46 and Ile117 which are missing in the human dUTPase (Hampton et al., 2011). Thus, structural modifications in trytil group should be further explored in order to improve the interactions with the hydrophobic pocket and, consequently, to help the design of novel potent and selective *Pf*dUTPase inhibitors. The CoMFA and CoMSIA electrostatic contour maps also show that electropositive groups in sugar moiety and uracil group are favorable for inhibition and selectivity (**Figures 3D, 4B,E**). Additionally, these maps show that electronegative groups near the region of the oxygen atom of the pentose sugar are favorable for *Pf*dUTPase selectivity (**Figure 4B**), while electronegative groups near the linker between the trityl group and the sugar moiety (**Figures 3B,D, 4E**) are unfavorable for both inhibition and selectivity.

The best individual HQSAR, CoMFA, and CoMSIA models were combined in a consensus approach (Supplementary Table S5). Thus, one consensus model for inhibition of *Pf*dUTPase and another for selectivity were built. The external validation of the consensus models was performed using the same external evaluation set and metrics used for individual QSAR models. The statistical characteristics of the consensus models are presented in **Table 1**. Both models showed good external predictivity (*Q*^2^_ext_ = 0.85 and 0.75; RMSEP = 0.40).

**Table 1:** Statistical characteristics of consensus QSAR models for *Pf*dUTPase inhibition and selectivity.

Model	*Q*^2^_ext_	RMSEP
Consensus – *Pf*dUTPase Inhibition^∗^	0.85	0.40
Consensus – Selectivity^∗^	0.75	0.40

*^∗^Consensus of the best individual HQSAR, CoMFA and CoMSIA models; Q^*2*^_*ext*_: determination coefficient for external set; RMSEP, root mean-square error of prediction.*

### Virtual Screening

The virtual screening of new potential *Pf*dUTPase inhibitors was performed on Hit2Lead library of ChemBridge database by prediction of activity and selectivity of the compounds through the developed and validated consensus QSAR models. Each consensus prediction was obtained by the arithmetic mean of the predictions from the best individual HQSAR, CoMFA, and CoMSIA models (Supplementary Table S6). All duplicates or compounds used to generate the models were excluded. Finally, the following criteria were used for selection of the virtual hits: (i) compounds should have the highest predicted potency against *Pf*dUTPase (predicted p*K*_i_) and (ii) the predicted selectivity (*S*) should be greater than zero. At the end of this process, five virtual hits were chosen for experimental evaluation.

Inadequate ADMET properties contribute to high failure rates in late stages of drug development. The early prediction and optimization of such properties can help the reduction of late-stage failures and expenses (van de Waterbeemd and Gifford, 2003; Sanders et al., 2017). In this study, the five virtual hits were evaluated by predicting/analyzing a panel of properties including log*P* and log*S*, oral acute toxicity in rodents (Filimonov et al., 2004; Lagunin et al., 2009, 2011), carcinogenicity (Cheng et al., 2012), and binding affinity to hERG (Braga et al., 2015) (**Table 2**). All molecules were predicted as non-carcinogenic and non-blockers of hERG channel. Only LabMol-143 and LabMol-146 were predicted as positive for acute oral toxicity. LabMol-142 presented a high calculated logP (7.3), while the remaining hits presented log*P* below or slightly above 5.

**Table 2:** Chemical structures, predicted potency against *Pf*dUTPase, predicted selectivity, and some calculated ADMET properties of the virtual hits.

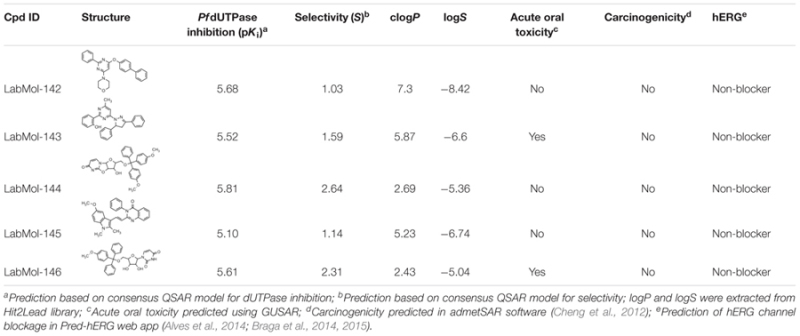

### Experimental Evaluation of Selected Compounds on *P. falciparum* Multi-Drug-Resistant and Sensitive Strains, and on *P. berghei* Sexual Stages

The five virtual hits selected were evaluated *in vitro* against asexual blood-stages of *P. falciparum* multi-drug-resistant (W2) and sensitive (3D7) strains. The half maximal inhibitory concentrations (IC_50_) for each compound (**Table 3**) indicate that three compounds (LabMol-144, LabMol-145, and LabMol-146) were more potent at inhibiting parasite growth, showing activity in submicromolar range against both 3D7 and W2 strains. Furthermore, the cytotoxicity was measured in mammalian COS7 cells. LabMol-144 and LabMol-146 showed promising results in terms of selectivity (SI = 11.7 and 6.7, respectively; **Table 3**).

**Table 3:** *In vitro* evaluation of selected hits against asexual blood stage of *P. falciparum* 3D7 and W2 strains, cytotoxicity on mammalian cells (COS7), selectivity index, and inhibition of ookinete stage of *P. berghei*.

Cpd ID	IC_50_ 3D7 (μM)	IC_50_ W2 (μM)	CC_50_ COS7 (μM)	SI	% conversion inhibition (10 μM)
LabMol-142	>40	>40	>100	ND	10.2 ± 11.9
LabMol-143	>40	>40	>200	ND	0
LabMol-144	7.1 ± 2.53	4.23 ± 1.18	81.7 ± 25.7	11.7	44.6 ± 2.4
LabMol-145	17.1 ± 16.2	15.3 ± 3.29	46.0 ± 13.4	2.7	13.2 ± 24.0
LabMol-146	6.1 ± 1.95	3.20 ± 2.12	52.0 ± 16.4	6.7	7.3 ± 7.3
Chloroquine	0.011 ± 0.0006	0.181 ± 0.027	–	–	–
Pyrimethamine	0.044 ± 0.009	14.7 ± 3.94	–	–	–

*IC_*50*_ 3D7: half maximal inhibitory concentration on 3D7 strain; IC_*50*_ W2: half maximal inhibitory concentration on W2 strain; CC_*50*_ COS7: half maximal cytotoxic concentration on COS7 cells; SI, selectivity index calculated between CC_*50*_ on COS7 and IC_*50*_ in 3D7 strain. The data are expressed as mean ± SD of three independent assays.*

The five compounds were also tested against *P. berghei* sexual stages using *in vitro* gametocyte to ookinete conversion assays (**Table 3**). LabMol-144, a promising selected compound in terms of IC_50_ and SI against asexual stages and mammalian cells, showed inhibition of 44.6% of ookinete formation relative to control. Although the IC_50_ range of LabMol-144 and LabMol-146 are still far from that of chloroquine and pyrimethamine (**Table 3**), these compounds represent good starting points for further optimization studies and development of new antimalarial drugs. In addition, drug development based on LabMol-144 may also lead to new antimalarials with transmission blocking activity and new mechanism of action.

The two most promising compounds, LabMol-144 and LabMol-146, are similar to the most potent compound from the training set (cpd. 1) used for developing QSAR models (*T*_c_ of 0.72 and 0.84, respectively, Supplementary Table S6). However, LabMol-144 presents some differences in relation to compound 1. As demonstrated on **Figures 2–4**, and based on previous reports on literature, the presence of hydrophobic groups on trytil region is favorable for both activity and selectivity against *Pf*dUTPase (Whittingham et al., 2005; Hampton et al., 2011; Ojha and Roy, 2013). Thus, LabMol-144 can be a potent and selective inhibitor of *Pf*dUTPase due to the addition of two methoxy substituents on trytil group, which can contribute for improved affinity to the hydrophobic binding pocket of the enzyme. Other modifications in LabMol-144 in comparison to compound 1 are the presence of the oxazolidine ring between the sugar moiety and uracil ring, and the substitution of nitrogen by oxygen on the linker between the sugar moiety and the trytil group.

**FIGURE 5:**
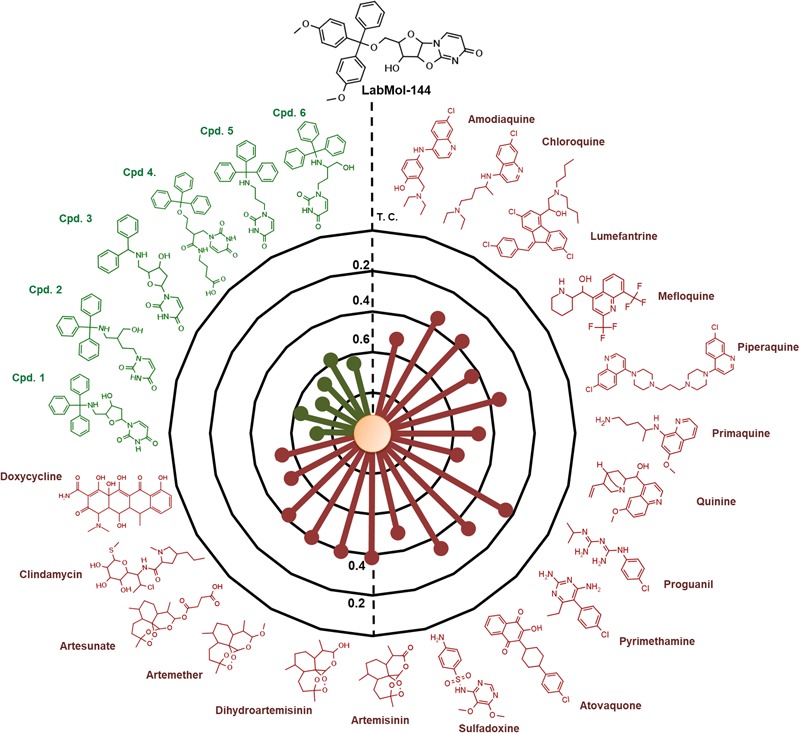
Radial plot showing the similarity of the most promising compound discovered (LabMol-144) compared to known antimalarial drugs (red) and six of the most potent inhibitors of *Pf*dUTPase from the dataset used for QSAR modeling (green). The similarity was calculated using Tanimoto coefficient (*T*_c_) and MACCS structural key fingerprints.

LabMol-144 has higher similarity to the most potent inhibitors of *Pf*dUTPase from the training set (compounds 1 to 6, **Figure 5**) *T*_c_ = 0.58–0.72, and it has a very low similarity to the currently used antimalarial drugs, *T*_c_ = 0.23–0.54 (**Figure 5**). Added to the fact that LabMol-144 showed similar activity against sensitive and multidrug resistant strains of *P. falciparum*, this further suggests that the mode of action of nucleosides and their derivatives is different from current antimalarials. This is particularly important considering parasite resistance in natural settings. Therefore, inhibitors of *Pf*dUTPase, a target different from the other test antimalarials, could overcome cross-resistance phenomena, and are very promising scaffolds to be explored as new antimalarial drugs. Certainly, the activity of compounds could be caused not only by *Pf*dUTPase inhibition but by different mechanisms of action. However, to explore this, further *in vitro* enzymatic studies should be performed. Exploring other mechanisms of action is out of the scope of this paper and should be considered in the next steps of the project.

### Molecular Docking

**FIGURE 6:**
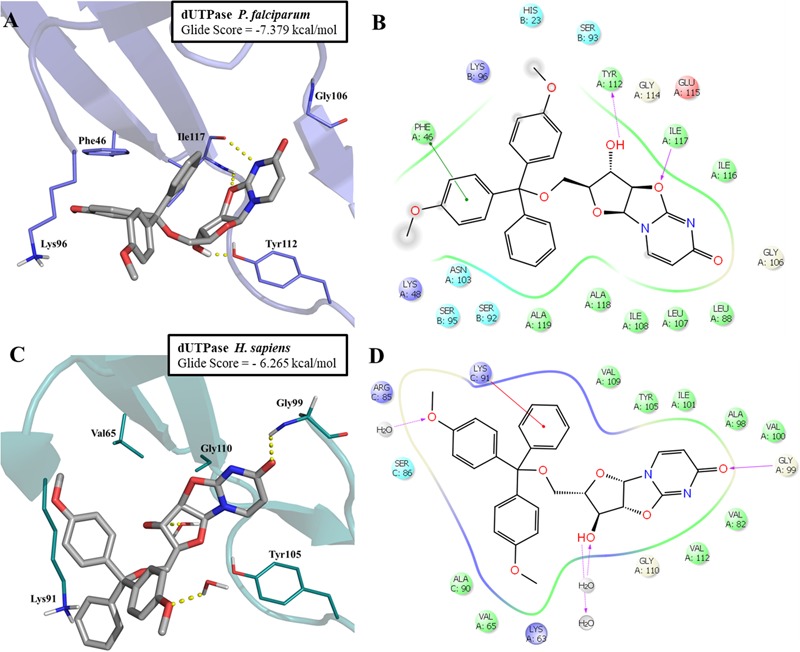
Molecular docking of LabMol-144 in dUTPase of *P. falciparum* **(A,B)** and human **(C,D)**. In 3D representation **(A,C)**, hydrogen bonds are presented as yellow dashed lines. In 2D interaction diagrams **(B,C)**, hydrogen bonds are presented as magenta arrows and hydrophobic interactions as red lines.

The most promising compound (LabMol-144, IC_50_ = 4.23 μM against W2 strain, and highest predicted pIC_50_ = 5.81 against the parasite enzyme) was docked in *Pf*dUTPase and *Hs*dUTPase in order to compare the binding modes and to analyze how differences between the human and parasite enzymes can be explored for the design of selective inhibitors. The docking studies suggested a higher affinity of LabMol-144 to *Pf*dUTPase. The Glide Score on *Pf*dUTPase was -7.38 kcal/mol (**Figure 6A**) and -6.26 kcal/mol on *Hs*dUTPase (**Figure 6C**). After the docking, we performed MM-GBSA calculations to obtain the free energy of binding, in order to compare the affinities of the compounds. The results are available on Supplementary Table S7. These results suggested that LabMol-144 has a higher affinity to *Pf*dUTPase, with a twice higher affinity toward the parasitic enzyme in comparison to the human ortholog (estimated ΔG of binding of -107.8 and -52.8, respectively).”

As demonstrated on **Figures 6A,B**, the parasitic enzyme has the amino acid residues Phe46 and Ile117 in the hydrophobic region of the active site, while the human counterpart has Val65 and Gly110, respectively (**Figures 6C,D**). The presence of Phe46 in *Pf*dUTPase is responsible for an additional π–π stacking interaction with one ring from trytil group, while Ile117 can perform two hydrogen bonds with uracil and oxazolidine rings. These two hydrogen bonds contribute to the exposure of a hydroxyl group to Tyr112, allowing the molecule to stablish an additional hydrogen bond with this residue (**Figure 6A**). The absence of Phe46 and Ile117 on the human enzyme (**Figure 6C**) results in a weaker affinity for Labmol-144. In *Hs*dUTPase, there are no interactions with Val65 and Gly110, and consequently, no hydrogen bond with Tyr105. The main interactions with *Hs*dUTPase are the hydrogen bonds with Gly99 and two structural water molecules (**Figures 6C,D**).

These results corroborate with our QSAR contribution and contour maps and also with previous studies (Whittingham et al., 2005; Hampton et al., 2011; Ojha and Roy, 2013), highlighting the differences between human and parasite enzymes, and the importance of hydrophobic interactions with trytil group for increased potency and selectivity. In future studies, we aim to perform enzymatic assays against human and plasmodial enzymes aiming to confirm the findings observed here. Furthermore, the *in vitro* results against multi-drug and sensitive *P. falciparum* strains and inhibition of *P. berghei* ookinete formation are indicative that LabMol-144 is an attractive scaffold for further hit-to-lead optimization studies for the development of new antimalarials with transmission blocking activity.

## Conclusion

In this work, we developed robust and externally predictive consensus QSAR models, merging 2D- (HQSAR) and 3D-QSAR (CoMFA and CoMSIA) models for prediction of inhibition and selectivity against *Pf*dUTPase. The QSAR models were applied for virtual screening of the ChemBridge database and allowed the selection of five new potential selective inhibitors of *Pf*dUTPase. The virtual hits were tested *in vitro* against sensitive (3D7) and multidrug-resistant (W2) strains of *P. falciparum*. Two compounds, LabMol-144 and LabMol-146, showed promising activity against both strains of *P. falciparum* and present chemical scaffolds very dissimilar from current antimalarial drugs. Thus, inhibitors of *Pf*dUTPase could be a good alternative for antimalarial drug combination. In addition, compound LabMol-144 showed potent *in vitro* inhibition of *P. berghei* ookinete formation, demonstrating that this compound is active against multiple parasite stages and, therefore, optimization based on this compound may also lead to new antimalarials with transmission blocking activity. In future studies, we aim to perform enzymatic assays against parasite and human enzymes. Furthermore, we aim to perform hit-to-lead optimization through structural modifications on the discovered scaffolds, based on the information gathered from the QSAR contribution and contour maps, aiming at designing new antimalarial drugs with transmission-blocking activity.

## Author Contributions

Each author has contributed significantly to this work. ML and CM-F contributed equally in the design, performing the computational experiments, and writing the paper. ML, CM-F, BN, VA, RB, PC, and CA conceived and designed the experiments. ML, CM-F, BN, VA, and RB performed the computational experiments. GC, FC, PC, JC, and DB performed the experimental assays. ML, CM-F, GC, FC, PC, EM, JC, and DB analyzed the data. ML, CM-F, GC, BN, EM, DB, and CA wrote the paper. All authors read, edited, and approved the final manuscript.

## Conflict of Interest Statement

The authors declare that the research was conducted in the absence of any commercial or financial relationships that could be construed as a potential conflict of interest. The handling Editor declared a shared affiliation, though no other collaboration, with one of the authors DB.

## Footnotes

1 http://www.chemaxon.com

2 http://www.eyesopen.com

3 http://www.hit2lead.com/

4 http://cactus.nci.nih.gov/chemical/apps/cap

5 https://omictools.com/admetsar-tool

6 http://labmol.com.br/predherg/
