# Supplementary material for: QSAR-Driven Design and Discovery of Novel Compounds With Antiplasmodial and Transmission Blocking Activities
Source: Front Pharmacol. 2018 Mar 6;9:146. doi: 10.3389/fphar.2018.00146 (PMC5845645; doi:10.3389/fphar.2018.00146)
Supplement: Supplementary file 1 [file Table_1.docx]

Supplementary Material

QSAR-Driven Design and Discovery of Novel Compounds with Antiplasmodial and Transmission Blocking Activities

**Marilia N. Nascimento Lima^1§^, Cleber C. Melo-Filho^1§^, Gustavo C. Cassiano^2^, Bruno J. Neves^1,3^, Vinicius M. Alves^1^, Rodolpho C. Braga^1^, Pedro V.L. Cravo^4^, Eugene N. Muratov^5,6^, Juliana Calit^7^, Daniel Y. Bargieri^7^, Fabio T.M. Costa^2^, Carolina Horta Andrade^1,2*^**

^1^LabMol - Laboratory for Molecular Modeling and Drug Design, Faculty of Pharmacy, Federal University of Goiás, Goiânia, Brazil

^2^Laboratory of Tropical Diseases – Prof. Dr. Luiz Jacintho da Silva, Department of Genetics, Evolution, Microbiology and Immunology, Institute of Biology, UNICAMP, Campinas, SP, Brazil

^3^Laboratory of Cheminformatics, University Center of Anápolis/UniEVANGELICA, Anápolis, Brazil

^4^Global Health and Tropical Medicine Centre (GHTM), Unidade de Parasitologia Médica, Instituto de Higiene e Medicina Tropical (IHMT), Universidade Nova de Lisboa, Lisboa, Portugal.

^5^Laboratory for Molecular Modeling, Division of Chemical Biology and Medicinal Chemistry, Eshelman School of Pharmacy, University of North Carolina, Chapel Hill, NC, United States

^6^Department of Chemical Technology, Odessa National Polytechnic University, Odessa, Ukraine

^7^Department of Parasitology, Institute of Biomedical Sciences, University of São Paulo, São Paulo, Brazil

^§^ These authors have equally contributed

*** Correspondence:** Carolina Horta Andrade: [carolina@ufg.br](mailto:carolina@ufg.br)

# Supplementary Figures and Tables

## Supplementary Figures


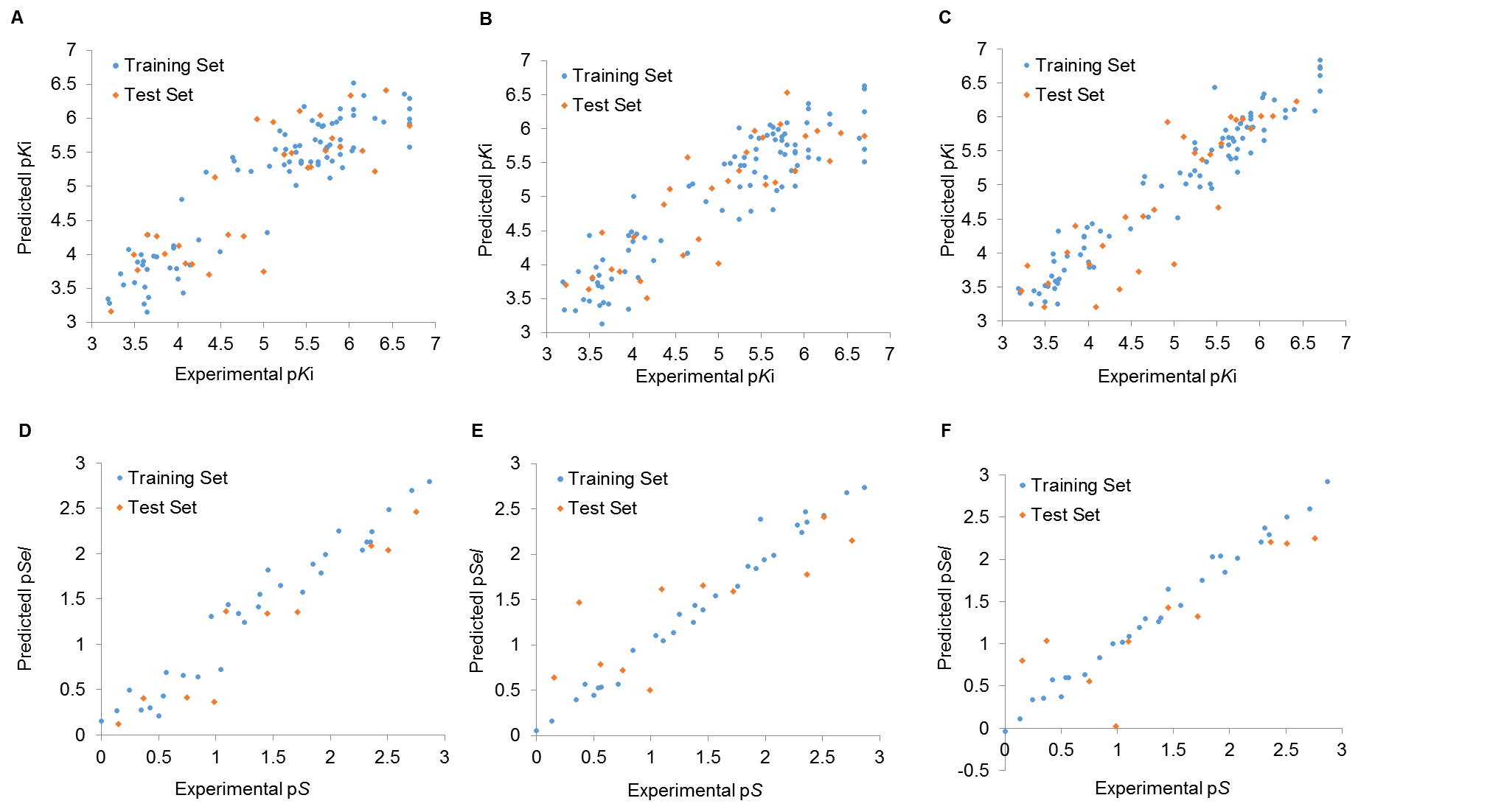
**Figure S1.** Experimental *versus* predicted biological activity of the best *Pf*dUTPase HQSAR **(A)**, *Pf*dUTPase CoMFA **(B)**, *Pf*dUTPase CoMSIA **(C),** *Sel* HQSAR **(D)**, *Sel* CoMFA **(E)** and *Sel* CoMSIA **(F)** models. The blue circles indicate the training set compounds and orange diamonds the test set compounds.

## Supplementary Tables

**Table S1.** Chemical structures and corresponding *Pf*dUTPase inhibition constant **(**p*Ki*) and selectivity (*S)* of the dataset used for QSAR model building and validation.

| **Compound** | **Structure** | **PfdUTPase inhibition (p*Ki*)** | **Selectivity**  **(*S*)** |
| --- | --- | --- | --- |
| 1 | 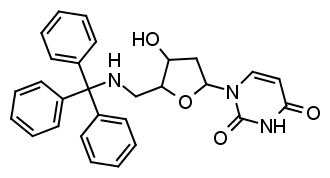 | 6.70 | 2.36 |
| 2 | 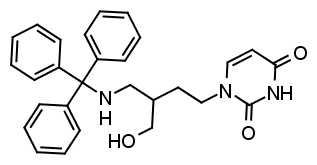 | 6.70 | 1.45 |
| 3 | 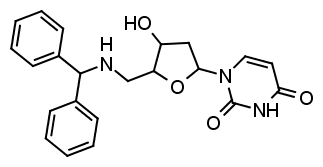 | 6.70 | - |
| 4 | 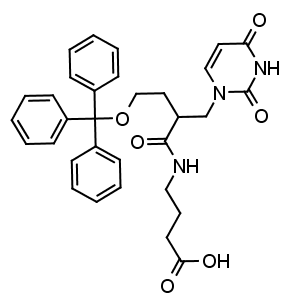 | 6.70 | - |
| 5 | 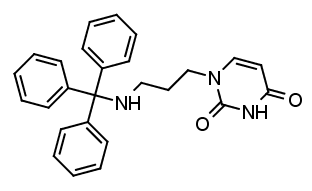 | 6.70 | 0.84 |
| 6 | 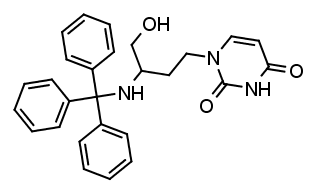 | 6.70 | 1.45 |
| 7 | 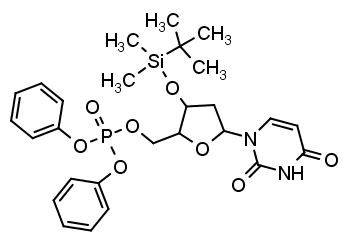 | 6.70 | 2.36 |
| 8 | 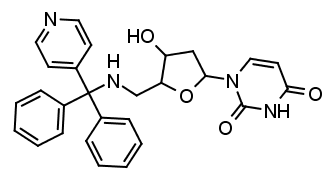 | 6.64 | - |
| 9 | 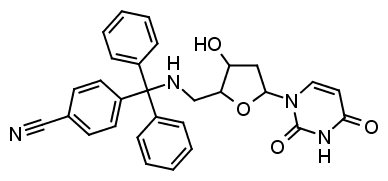 | 6.43 | 2.76 |
| 10 | 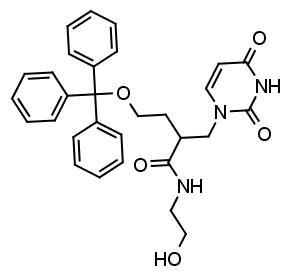 | 6.40 | - |
| 11 | 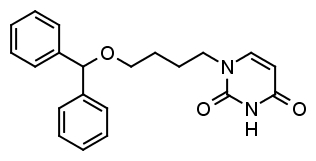 | 6.30 | 1.99 |
| 12 | 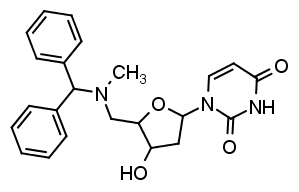 | 6.30 | 1.72 |
| 13 | 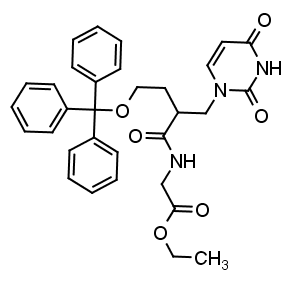 | 6.30 | - |
| 14 | 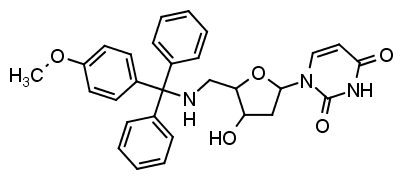 | 6.17 | 2.71 |
| 15 | 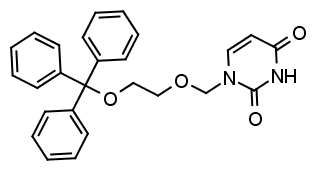 | 6.15 | 1.38 |
| 16 | 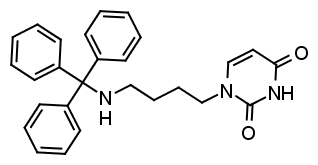 | 6.05 | - |
| 17 | 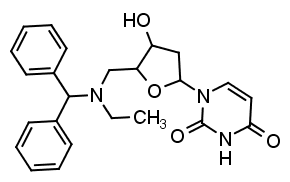 | 6.05 | 1.56 |
| 18 | 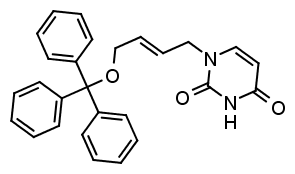 | 6.05 | - |
| 19 | 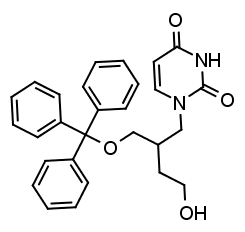 | 6.05 | 1.37 |
| 20 | 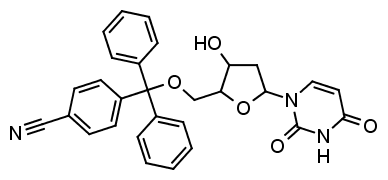 | 6.03 | 2.87 |
| 21 | 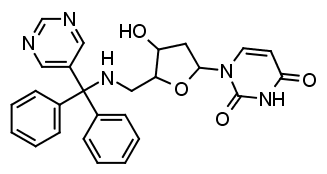 | 6.01 | - |
| 22 | 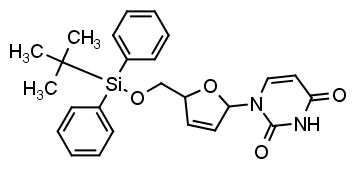 | 5.92 | - |
| 23 | 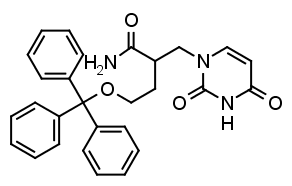 | 5.89 | - |
| 24 | 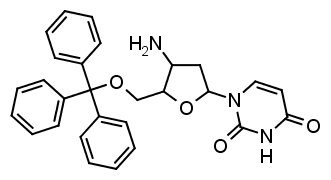 | 5.89 | - |
| 25 | 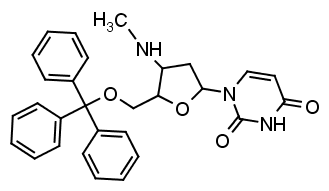 | 5.89 | - |
| 26 | 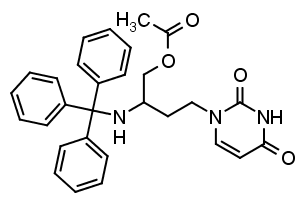 | 5.89 | 1.76 |
| 27 | 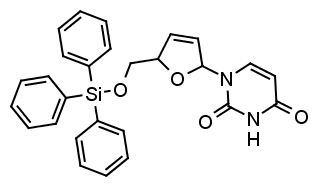 | 5.89 | - |
| 28 | 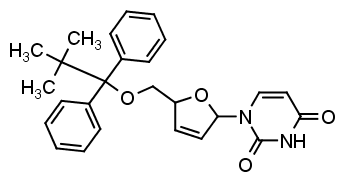 | 5.89 | - |
| 29 | 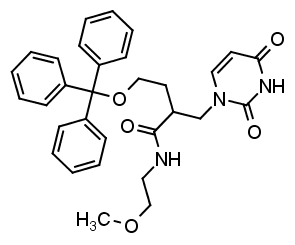 | 5.85 | - |
| 30 | 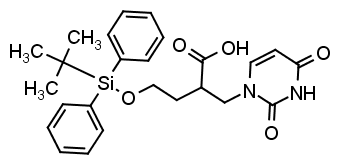 | 5.80 | - |
| 31 | 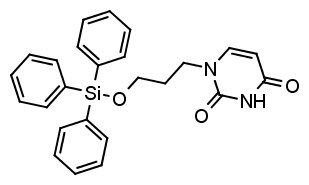 | 5.80 | 1.19 |
| 32 | 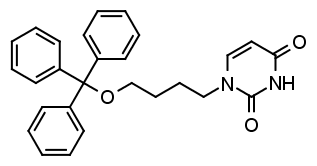 | 5.80 | - |
| 33 | 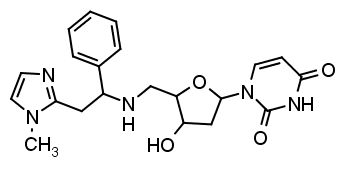 | 5.77 | - |
| 34 | 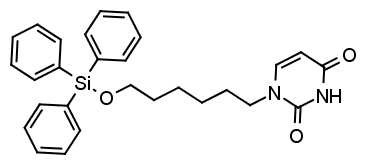 | 5.77 | 2.35 |
| 35 | 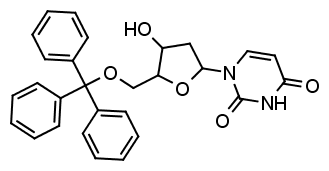 | 5.74 | 0.98 |
| 36 | 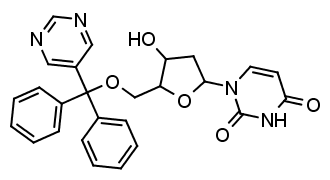 | 5.74 | - |
| 37 | 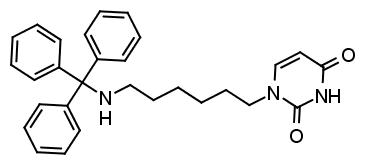 | 5.74 | - |
| 38 | 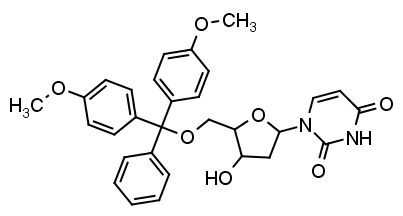 | 5.72 | - |
| 39 | 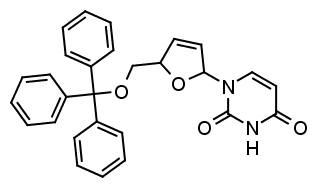 | 5.72 | 1.92 |
| 40 | 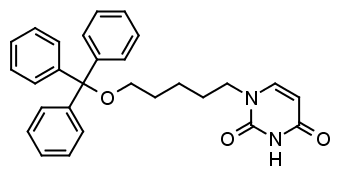 | 5.70 | - |
| 41 | 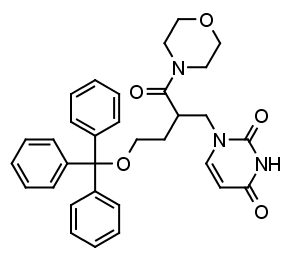 | 5.68 | - |
| 42 | 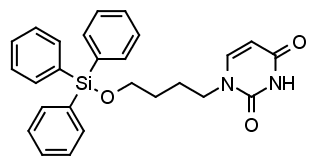 | 5.66 | - |
| 43 | 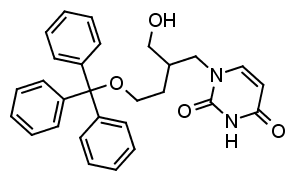 | 5.66 | -2.07 |
| 44 | 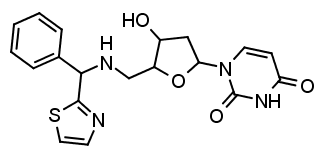 | 5.64 | - |
| 45 | 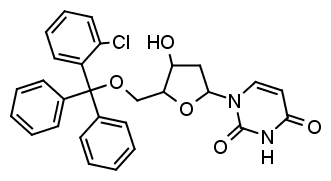 | 5.64 | - |
| 46 | 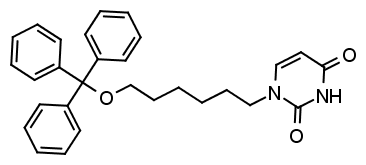 | 5.64 | 2.32 |
| 47 | 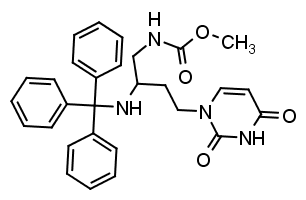 | 5.60 | - |
| 48 | 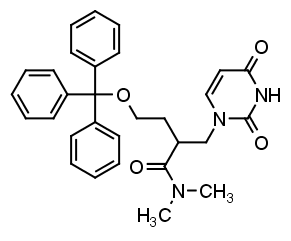 | 5.57 | - |
| 49 | 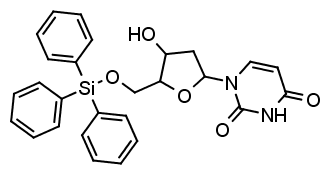 | 5.55 | 2.51 |
| 50 | 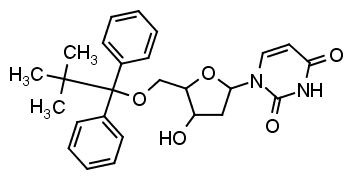 | 5.55 | 2.51 |
| 51 | 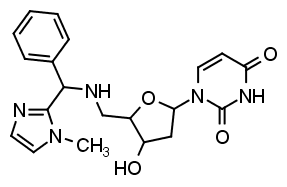 | 5.52 | - |
| 52 | 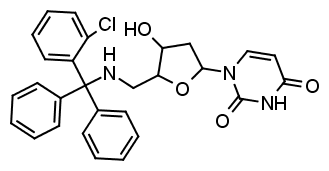 | 5.47 | 1.84 |
| 53 | 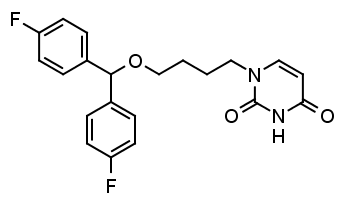 | 5.44 | 1.11 |
| 54 | 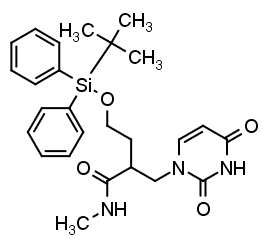 | 5.44 | - |
| 55 | 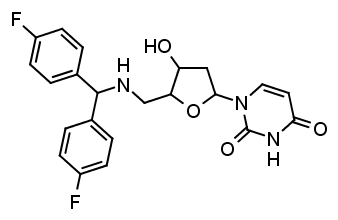 | 5.42 | - |
| 56 | 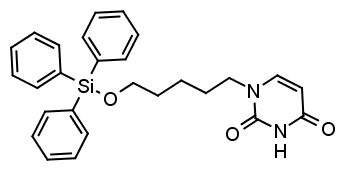 | 5.42 | - |
| 57 | 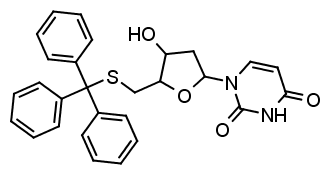 | 5.38 | - |
| 58 | 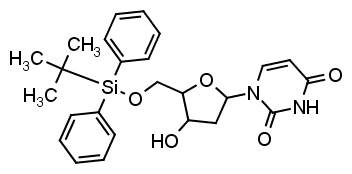 | 5.38 | 2.28 |
| 59 | 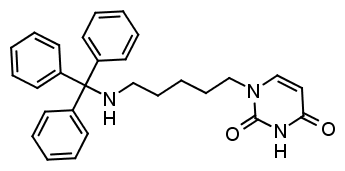 | 5.37 | - |
| 60 | 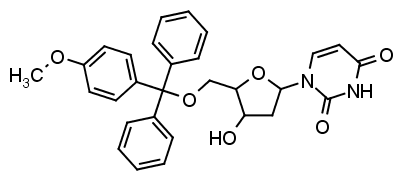 | 5.33 | - |
| 61 | 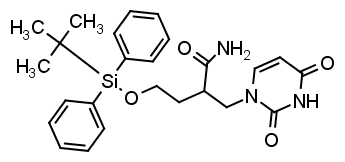 | 5.30 | - |
| 62 | 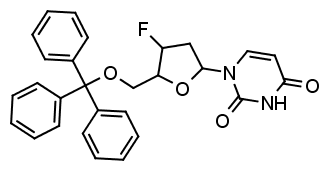 | 5.30 | 1.96 |
| 63 | 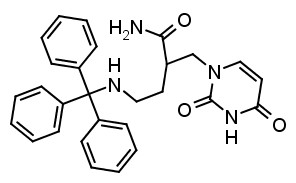 | 5.26 | 1.25 |
| 64 | 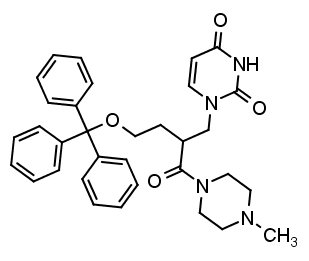 | 5.25 | - |
| 65 | 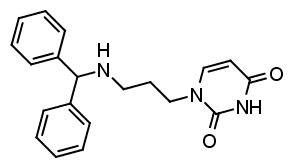 | 5.24 | 0.24 |
| 66 | 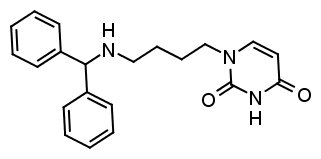 | 5.24 | 1.04 |
| 67 | 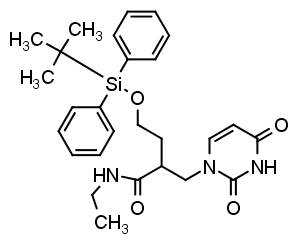 | 5.24 | - |
| 68 | 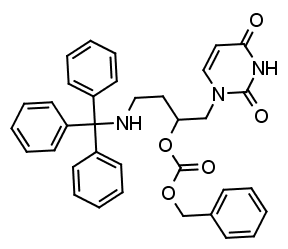 | 5.19 | - |
| 69 | 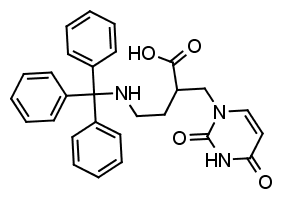 | 5.14 | 1.10 |
| 70 | 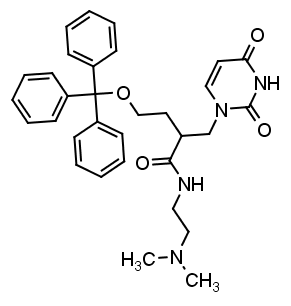 | 5.11 | - |
| 71 | 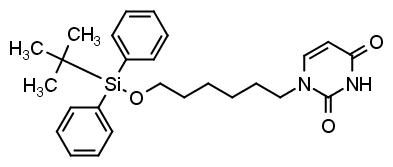 | 5.07 | - |
| 72 | 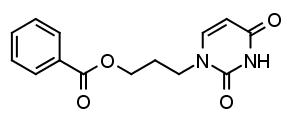 | 5.04 | 1.99 |
| 73 | 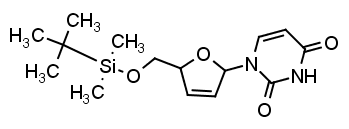 | 5.00 | 0.75 |
| 74 | 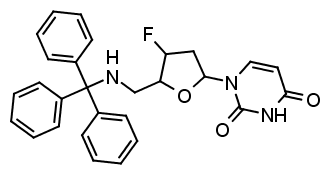 | 4.92 | - |
| 75 | 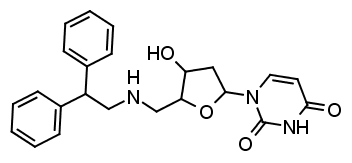 | 4.85 | 0.37 |
| 76 | 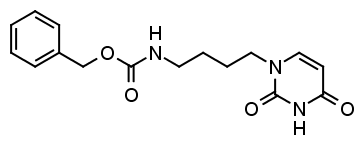 | 4.77 | - |
| 77 | 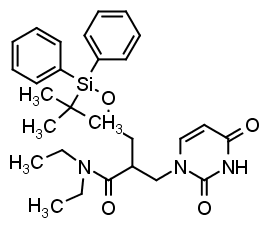 | 4.70 | - |
| 78 | 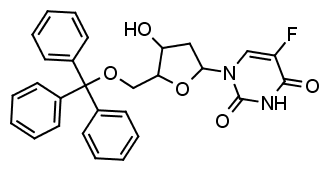 | 4.66 | - |
| 79 | 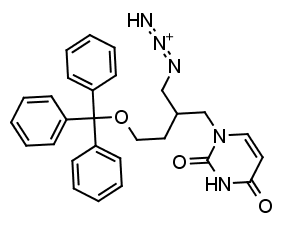 | 4.64 | - |
| 80 | 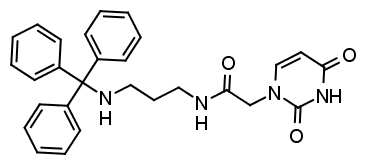 | 4.64 | - |
| 81 | 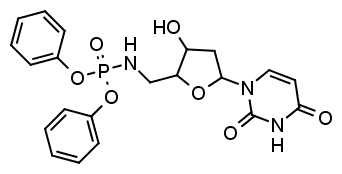 | 4.59 | - |
| 82 | 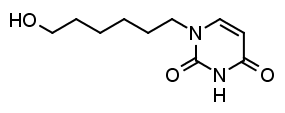 | 4.49 | - |
| 83 | 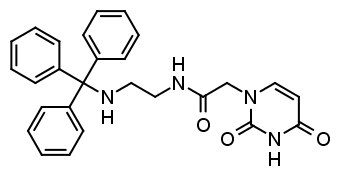 | 4.43 | - |
| 84 | 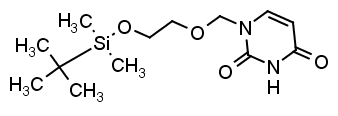 | 4.36 | 0.16 |
| 85 | 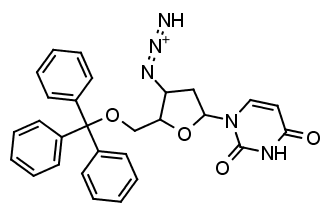 | 4.33 | - |
| 86 | 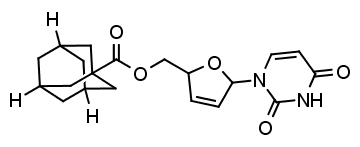 | 4.24 | 0.71 |
| 87 | 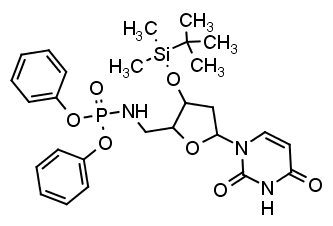 | 4.17 | 0.54 |
| 88 | 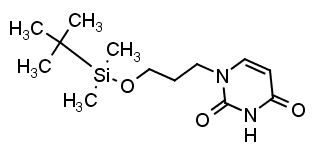 | 4.14 | 0 |
| 89 | 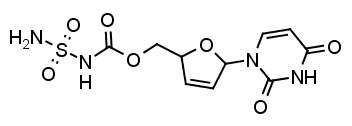 | 4.09 | 0.99 |
| 90 | 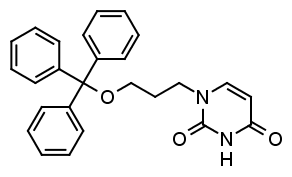 | 4.06 | 0.56 |
| 91 | 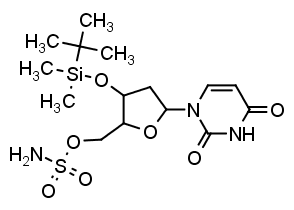 | 4.06 | 0.57 |
| 92 | 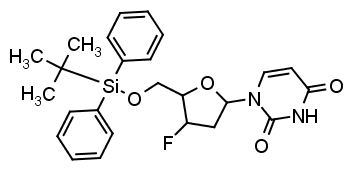 | 4.05 | 0.96 |
| 93 | 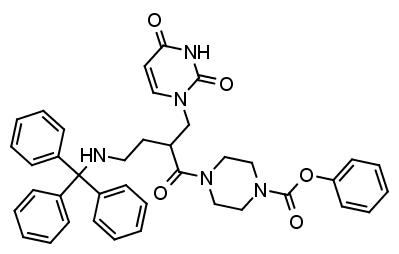 | 4.01 | - |
| 94 | 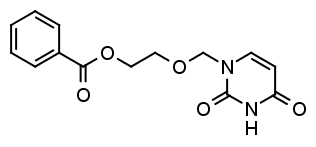 | 4.01 | 0.50 |
| 95 | 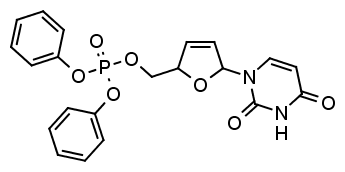 | 4.00 | - |
| 96 | 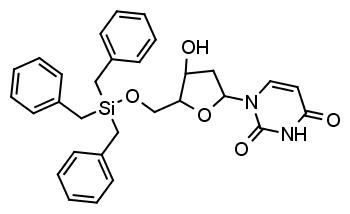 | 3.99 | - |
| 97 | 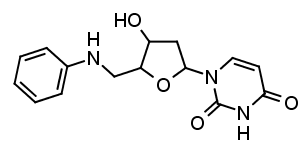 | 3.95 | - |
| 98 | 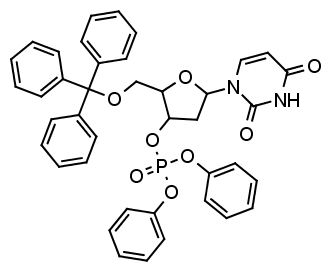 | 3.95 | - |
| 99 | 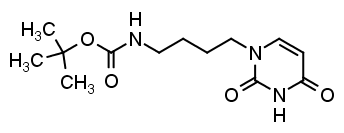 | 3.95 | - |
| 100 |  | 3.91 | - |
| 101 |  | 3.85 | - |
| 102 |  | 3.75 | 0.42 |
| 103 |  | 3.75 | - |
| 104 |  | 3.72 | - |
| 105 |  | 3.70 | - |
| 106 |  | 3.66 | - |
| 107 |  | 3.65 | 0.34 |
| 108 |  | 3.64 | - |
| 109 |  | 3.64 | - |
| 110 |  | 3.64 | - |
| 111 |  | 3.62 | - |
| 112 |  | 3.61 | - |
| 113 |  | 3.60 | - |
| 114 |  | 3.59 | 0.14 |
| 115 |  | 3.57 | - |
| 116 |  | 3.53 | - |
| 117 |  | 3.53 | - |
| 118 |  | 3.50 | - |
| 119 |  | 3.50 | - |
| 120 |  | 3.49 | - |
| 121 |  | 3.43 | - |
| 122 |  | 3.37 | - |
| 123 |  | 3.33 | - |
| 124 |  | 3.29 | - |
| 125 |  | 3.22 | - |
| 126 |  | 3.20 | - |
| 127 |  | 3.19 | - |

**Table S2.** Statistical characteristics of the best HQSAR models for *Pf*dUTPase inhibition and selectivity.

| ***Pf*dUTPase Inhibition** | | | | | | | | | |
| --- | --- | --- | --- | --- | --- | --- | --- | --- | --- |
| **Model** | **Frag. distinction** | **size** | ***q^2^_LOO_*** | ***r^2^*** | ***SEE*** | ***HL*** | ***N*** | ***Q^2^_ext_*** | ***d*** |
| 1 | A/B/C | 5-8 | 0.69 | 0.84 | 0.42 | 809 | 6 | 0.71 | 2.03 |
| **2** | **A/C** | **5-8** | **0.70** | **0.84** | **0.42** | **997** | **6** | **0.71** | **1.99** |
| 3 | C | 5-8 | 0.71 | 0.84 | 0.42 | 673 | 6 | 0.69 | 2.18 |
| **Selectivity** | | | | | | | | | |
| **Model** | **Frag. distinction** | **size** | ***q^2^_LOO_*** | ***r^2^*** | ***SEE*** | ***HL*** | ***N*** | ***Q^2^_ext_*** | ***d*** |
| 1 | C/Ch/Da | 4-7 | 0.61 | 0.91 | 0.28 | 61 | 6 | 0.76 | 2.22 |
| 2 | **B/C** | **7-10** | **0.61** | **0.95** | **0.21** | **97** | **6** | **0.83** | **2.02** |
| 3 | C | 2-5 | 0.58 | 0.65 | 0.49 | 53 | 1 | 0.46 | 2.15 |

*q^2^_LOO_*, leave-one-out cross-validated determination coefficient; *r^2^*, non-cross-validated determination coefficient; *SEE*, standard error of estimate; *HL,* hologram length; *N*, optimal number of latent variables in PLS analysis; *Q^2^_ext_*, determination coefficient for external set; *d*, Durbin-Watson coefficient. Fragment distinctions: A, atoms; B, bonds; C, connectivity; Ch, Chirality; DA, donor and acceptor. The best HQSAR models are highlighted in bold font.

**Table S3.** Statistical characteristics of the best CoMFA models for *Pf*dUTPase inhibition and selectivity.

| ***Pf*dUTPase Inhibition** | | | | | | | |
| --- | --- | --- | --- | --- | --- | --- | --- |
| **Model** | **Alignment** | **SDC** | ***q^2^_LOO_*** | ***r^2^*** | ***SEE*** | ***Q^2^_ext_*** | ***d*** |
| 1 | Suflex-Sim^a^ | 0.5 | 0.66 | 0.94 | 0.28 | 0.64 | 2.32 |
| **2** | **ROCS^a^** | **0.7** | **0.63** | **0.84** | **0.43** | **0.75** | **1.87** |
| 3 | Docking^a^ | 0.9 | 0.67 | 0.74 | 0.54 | 0.63 | 2.04 |
| **Selectivity** | | | | | | | |
| **Model** | **Alignment** | **SDC** | ***q^2^_LOO_*** | ***r^2^*** | ***SEE*** | ***Q^2^_ext_*** | ***d*** |
| **1** | **Surflex-Sim^a^** | **0.6** | **0.86** | **0.98** | **0.12** | **0.61** | **1.99** |
| 2 | ROCS^a^ | 1.1 | 0.79 | 0.99 | 0.09 | 0.57 | 1.80 |
| 3 | Docking^a^ | 0.3 | 0.68 | 0.99 | 0.025 | 0.33 | 2.11 |

^a^Gasteiger-Hückel partial charges; ^b^AM1-BCC partial charges; SDC, standard deviation coefficient used for field focus; *q^2^_LOO_*, leave-one-out cross-validated determination coefficient; *r^2^*, non-cross-validated determination coefficient; *SEE*, standard error of estimate; *Q^2^_ext_*, determination coefficient for external set; *d*, Durbin-Watson coefficient. The best CoMFA models are highlighted in bold font

**Table S4.** Statistical characteristics of the best CoMSIA models for *Pf*dUTPase inhibition and selectivity.

| ***Pf*dUTPase Inhibition** | | | | | | | |
| --- | --- | --- | --- | --- | --- | --- | --- |
| **Model** | **Alignment** | **Molecular Fields** | ***q^2^_LOO_*** | ***r^2^*** | ***SEE*** | ***Q^2^_ext_*** | ***d*** |
| 1 | Suflex-Sim^a^ | S, H, D | 0.62 | 0.88 | 0.38 | 0.75 | 2.03 |
| **2** | **ROCS^b^** | **E, H** | **0.68** | **0.94** | **0.28** | **0.78** | **2.28** |
| 3 | Docking^b^ | SED | 0.590 | 0.32 | 0.91 | 0.64 | 2.70 |
| **Selectivity** | | | | | | | |
| **Model** | **Alignment** | **Molecular Fields** | ***q^2^_LOO_*** | ***r^2^*** | ***SEE*** | ***Q^2^_ext_*** | ***d*** |
| 1 | Surflex^a^ | SHA | 0.63 | 0.97 | 0.14 | 0.29 | 2.34 |
| **2** | **ROCS^b^** | **E, H, S** | **0.59** | **0.99** | **0.1** | **0.63** | **1.63** |
| 3 | Docking^b^ | HDA | 0.543 | 0.99 | 0.09 | -0.46 | 1.73 |

^a^Gasteiger-Hückel partial charges; ^b^AM1-BCC partial charges; *q^2^_LOO_*, leave-one-out cross-validated determination coefficient; *r^2^*, non-cross-validated determination coefficient; *SEE*, standard error of estimate; *Q^2^_ext_*, determination coefficient for external set; *d*, Durbin-Watson coefficient; Molecular Fields: A, acceptor; D, donor; E, electrostatic; H, hydrophobic; S, steric. The best CoMSIA models are highlighted in bold font.

**Table S5.** Statistical characteristics for the best individual and consensus models for *Pf*dUTPase inhibition and selectivity.

| *Pf*dUTPase inhibiton | | | | | | | |
| --- | --- | --- | --- | --- | --- | --- | --- |
| Model | Alignment | | *q^2^_LOO_* | | *r^2^* | *Q^2^_ext_* | *d* |
| HQSAR | - | | 0.70 | | 0.84 | 0.71 | 1.99 |
| CoMFA | ROCS^a^ | | 0.63 | | 0.83 | 0.75 | 1.86 |
| CoMSIA | Docking^b^ | | 0.68 | | 0.94 | 0.78 | 2.28 |
| Selectivity | | | | | | | |
| Model | Alignment | | *q^2^_LOO_* | | *r^2^* | *Q^2^_ext_* | *d* |
| HQSAR | - | | 0.61 | | 0.95 | 0.83 | 2.02 |
| CoMFA | Surflex-Sim^a^ | | 0.86 | | 0.98 | 0.61 | 1.99 |
| CoMSIA | ROCS^b^ | | 0.59 | | 0.99 | 0.63 | 1.63 |
| Consensus Models | | | | | | | |
| Model | | *Q^2^_ext_* | | RMSEP | | | |
| *Pf*dUTPase Inibition | | 0.85 | | 0.40 | | | |
| Selectivity | | 0.75 | | 0.40 | | | |

^a^Gasteiger-Hückel partial charges; ^b^AM1-BCC partial charges; *q^2^_LOO_*, leave-one-out cross-validated determination coefficient; *r^2^*, non-cross-validated determination coefficient; *Q^2^_ext_*, determination coefficient for external set; *d*, Durbin-Watson coefficient.

**Table S6.** Predictions of *Pf*dUTPase inhibition and selectivity for the five selected compounds in virtual screening.

| Cpd. | *Pf*dUTPase inhibition  (predicted p*Ki*) | | | | Selectivity  (predicted *S*) | | | | *Tc** |
| --- | --- | --- | --- | --- | --- | --- | --- | --- | --- |
|  | HQSAR | CoMFA | CoMSIA | Consensus | HQSAR | CoMFA | CoMSIA | Consensus |  |
| LabMol-142 | 5.13 | 5.21 | 6.14 | 5.68 | 0.98 | 0.96 | 1.09 | 1.03 | 0.65 |
| LabMol-143 | 5.15 | 4.57 | 6.84 | 5.52 | 1.98 | 1.32 | 1.47 | 1.59 | 0.49 |
| LabMol-144 | 5.56 | 5.59 | 6.29 | 5.81 | 3.51 | 2.35 | 2.06 | 2.64 | 0.72 |
| LabMol-145 | 5.01 | 4.08 | 6.20 | 5.10 | 1.00 | 1.18 | 1.24 | 1.14 | 0.53 |
| LabMol-146 | 5.54 | 5.42 | 5.87 | 5.61 | 2.93 | 2.09 | 1.92 | 2.31 | 0.84 |

^*^ Tanimoto coefficient between the virtual hits and compound 1 (most potent on dataset for QSAR modeling) calculated by MACCS structural key fingerprint similarity, implemented on CDK nodes extension for KNIME (Beisken et al., 2013; Berthold et al., 2007).

**Table S7.**Calulation of the free energy of binding, using MM-GBSA, for the five compounds selected after virtual screening.

| **Compound** | **MM-GBSA ΔG (Kcal/mol)**  ***Pf*dUTPase** | **MM-GBSA-ΔG (Kcal/mol)**  ***Hs*dUTPase** |
| --- | --- | --- |
|  |  |  |
| **LabMol-142** | -51.045 | -48.36 |
| **LabMol-143** | -66.31 | -40.07 |
| **LabMol-144** | -107.83 | -52.82 |
| **LabMol-145** | -46.12 | -40.75 |
| **LabMol-146** | -90.18 | -57.27 |

**References**

Beisken, S., Meinl, T., Wiswedel, B., de Figueiredo, L. F., Berthold, M., and Steinbeck, C. (2013). KNIME-CDK: Workflow-driven cheminformatics. *BMC Bioinformatics* 14, 257. doi:10.1186/1471-2105-14-257.

Berthold, M. R., Cebron, N., Dill, F., Gabriel, T. R., Kötter, T., Meinl, T., et al. (2007). KNIME: The Konstanz Information Miner. in *Studies in Classification, Data Analysis, and Knowledge Organization* (Springer).
